# Neuroprotection after infection-sensitized neonatal hypoxic-ischemic brain injury

**DOI:** 10.1186/2194-7791-2-S1-A11

**Published:** 2015-07-01

**Authors:** Hemmen Sabir, Damjan Osredkar, Elke Maes, Thomas Wood, Marianne Thoresen

**Affiliations:** Department of General Pediatrics, Neonatology and Pediatric Cardiology, Children's Hospital, Heinrich-Heine University Düsseldorf, Düsseldorf, Germany; Department of Physiology, Institute of Basic Medical Sciences, University of Oslo, Oslo, Norway

## Background

Therapeutic hypothermia (HT) is standard treatment after perinatal hypoxic-ischemic (HI) injury. However, around 50% of asphyxiated newborns still suffer poor outcome, some of which may have been exposed to perinatal infection prior to HI, as infection may increase vulnerability to HI injury.

## Aims

To develop an infection-sensitized newborn animal model of HI injury and to investigate whether HT is neuroprotective in this setting.

## Methods

Seven day old rat pups were injected either with vehicle (NaCl 0.9%) or E.coli lipopolysaccharide (LPS). Subsequently, they were exposed to left carotid ligation followed by global hypoxia inducing a unilateral HI injury. Pups were randomized to the following treatments: 1) Vehicle treated HI-pups receiving normothermia treatment (NT) (Veh-NT); 2) LPS treated HI-pups receiving NT treatment (LPS-NT); 3) vehicle treated HI-pups receiving HT treatment (Veh-HT); or 4) LPS treated HI-pups receiving HT treatment (LPS-HT). Relative area loss of the left/right hemisphere and the areas of hippocampi were measured after one-week survival.

## Results

Mean brain area loss in the Veh-NT group was 11.2%±14%. The brain area loss in LPS-NT pups was 29.8%±17%, being significantly higher than in the Veh-NT group (p = 0.002). The Veh-HT group had a significantly smaller brain area loss (5.4%±6%) when compared to Veh-NT group (p = 0.043). The LPS-HT group showed a brain area loss of 32.5%±16%, being significantly higher than in the Veh-HT group (p < 0.001). LPS-HT group also had significantly smaller size of the left hippocampus. LPS-sensitization significantly decreased the sizes of the right, unligated-hemispheres, independent of post-HI treatment.

## Conclusions

Therapeutic hypothermia is not neuroprotective in this infection-sensitized HI brain injury model. Lack of neuroprotection was particularly seen in the hippocampus. The exact mechanisms remain unclear and further investigation is needed to explain why a neuroprotective therapy loses its efficacy and how hypothermia may be optimized.

